# Assessment of a Mobile App by Adolescents and Young Adults With Cystic Fibrosis: Pilot Evaluation

**DOI:** 10.2196/12442

**Published:** 2019-11-21

**Authors:** Isa Rudolf, Katharina Pieper, Helga Nolte, Sibylle Junge, Christian Dopfer, Annette Sauer-Heilborn, Felix C Ringshausen, Burkhard Tümmler, Ute von Jan, Urs-Vito Albrecht, Jan Fuge, Gesine Hansen, Anna-Maria Dittrich

**Affiliations:** 1 Pediatric Pneumology, Allergology, and Neonatology Hannover Medical School Hannover Germany; 2 Biomedical Research in End-Stage and Obstructive Lung Disease Hannover German Centre for Lung Research Hannover Medical University Hannover Germany; 3 Haus Schutzengel Mukoviszidose eV Hannover Germany; 4 Department of Respiratory Medicine Hannover Medical University Hannover Germany; 5 Peter L Reichertz Institute for Medical Informatics Hannover Medical School Hannover Germany

**Keywords:** mobile phone, mobile phone app, mHealth, self-management, adolescence, cystic fibrosis

## Abstract

**Background:**

Cystic fibrosis (CF) continues to be the most common life-limiting chronic pulmonary disease in adolescents and young adults. Treatment of CF demands a high treatment time investment to slow the progression of lung function decline, the most important contributor to morbidity and mortality. Adherence is challenging in CF due to the high treatment burden and the lack of immediate health consequences in case of nonadherence. Lung function decline is particularly pronounced in the transition phase between 12 and 24 years of age. The improvement of self-management and self-responsibility and independence from parents and desire for normalcy are conflicting aspects for many adolescents with CF, which influence adherence to the time-consuming pulmonary therapy. Mobile health (mHealth) care apps could help to support self-management and independence and thereby reconcile seemingly conflicting goals to improve adherence, quality of life, and ultimately CF life expectancy.

**Objective:**

This study aimed to (1) assess user behavior and satisfaction among adolescents and young adults with CF over an observation period of three months using an mHealth app; (2) identify areas of improvement for this mHealth app; and (3) compare overall and disease-specific satisfaction, lung function, and anthropometry before and after using the mHealth app.

**Methods:**

A total of 27 adolescents and young adults with CF (age range 12-24 years, mean age 16 years, SD 3 years; 14 females, 11 males) used a free mHealth app for three months of whom 25 provided questionnaire data for analysis at the end of the study. Data collection was carried out using questionnaires on usage characteristics and life satisfaction, and standardized assessment of lung function and anthropometry.

**Results:**

The use of the reminder function for medication declined from 70% (15/21) of the participants at week 4 to 65% (13/20) at week 8 of the observation period. At the end of the study, only 17% (4/23) of the participants wanted to continue using the app. Nevertheless, 56% (14/25) of participants saw the mobile app as a support for everyday life. Potential improvements targeting hedonistic qualities were identified to improve mHealth app adherence. Comparisons of satisfaction with different life aspects hinted at improvements or stabilization for the subitem respiration and the subitem lack of handicap by CF, suggesting that app use might stabilize certain CF-specific aspects of the weighted satisfaction with life. Lung function and anthropometry were not affected consistently.

**Conclusions:**

Most of the patients did not want to continue using the app after the study period. Only a few CF-specific aspects of weighted life satisfaction were possibly stabilized by the mHealth app; clinical parameters were not affected. Adaptation of the functions to adolescent-specific needs could improve the long-term use and thus positively affect the disease course.

## Introduction

### Background

Cystic fibrosis (CF) is the most common systemic metabolic disease in Caucasians, affecting more than 70,000 patients worldwide, about 8000 of whom reside in Germany, where currently about 55% are in the adolescent or transition age, that is, between 12 and 29 years old [1]. Despite progress, CF remains life-limiting, mainly because of chronic pulmonary disease, with a high morbidity burden.

Treatment of CF demands one of the highest treatment time investments of all chronic diseases, which turns treatment adherence critical and vulnerable [2,3]. Treatment time investment in CF is high mainly because of the need for stringent, twice daily inhalational therapy coupled with physiotherapy techniques, but also because of the continuous need of enzyme replacement therapy with every fat-containing meal. Changes in everyday symptom load typically occur in the context of common colds and need to be judged by the patient for their severity, as they might necessitate treatment changes, that is, additional inhalations or antibiotics. Adherence in CF is particularly challenging, because of not only the high time investment but also the lack of immediate changes in health status in case of nonadherence, as disease progression is not immediately palpable in case of lung function decline.

In CF, as in all chronic diseases, the transition phase, that is, the period of life between approximately 12 and 24 years of age, constitutes the period in life when the most rapid loss of organ function can be observed [3], crucially setting the stage for future health [4]. A large proportion of loss of organ function is due to the psychological demands inherent to puberty. Adolescents afflicted by a chronic disease, which demands a high degree of treatment adherence, face the difficulty to meet the requirements of their disease, including the development of sufficient disease investment, in addition to the pubertal requirements to develop self-responsibility, self-determination, and independence [5]. During adolescence, patients with CF must learn to assess changes in everyday symptom load by themselves, to be able to take over this critical task from their parents. Disease investment because of a high load of therapy and the need to monitor health status versus parental independence and the desire for normalcy create conflicting situations for adolescents with chronic disease and turn adherence into a particularly vulnerable quality during this period of life [5-7]. Development of independent self-management techniques, a goal for many adolescents, is thus of an even higher priority in adolescents with chronic diseases such as CF.

The adolescent population, regardless of having a chronic disease, is difficult to approach, often coupled to a lack of attainability via conventional communication venues. Communication via mobile phones constitutes a promising venue in that respect. In Germany, in 2015, 90% of adolescents aged between 14 and 19 years owned a mobile phone, of whom 80% considered their mobile phone to be *very important* or *important* to them [8], providing proof to the common knowledge of high mobile phone affinity of this age group worldwide [9]. Currently available mobile apps offer novel approaches to support the daily therapy of patients with chronic diseases. On those lines, systematic reviews show promise in the use of mobile health (mHealth) apps to improve self-management for chronic diseases [10], also in the area of CF [7]. However, many of the existing mHealth apps are not disease specific [11], or do not provide all the desirable functions for the particular disease [12], although this is a typical demand in user-centered surveys among CF patients [9,11]. Development of CF-specific mHealth apps by pharmaceutical companies introduces a bias, possibly conflicting with patients’ actual needs [13]. Some comprehensive mHealth apps have been designed by CF patients themselves [14] or doctors involved in CF patients’ care [14-16], yet language and differences in medical systems provide barriers to extrapolation in different countries. Stringent scientific evaluation of mHealth apps, specifically for patients in the transition age, remains rare [9], even though studies have shown age-dependent differences in usage of mHealth apps [17].

### Hypothesis

The studies cited above indicate that rigorous analyses of mHealth app use in adolescents with CF are necessary when aiming at the development of supportive mHealth apps for this particular age group. We hypothesized that a positive assessment of a mobile phone app would lead to sustained use over time.

### Objectives

To address this hypothesis, we introduced a subgroup of our CF patients in our transition clinic to a mobile phone app, the KiOAPP [18], designed to support disease management. There are several assets in KiOAPP that influenced our choice of this specific app. As CF doctors, we consider self-management and communication essential to develop sustained adherence, regardless of patients’ age. KiOAPP addresses these aspects by a diary, communication venue, a medication plan, and reminder function. Moreover, the developers of KiOAPP claim age-specific tailoring, for example, by using youth-specific, less formal language, colorful and adaptable design, placing emphasis on user autonomy [19], which might be essential to improve user affinity and, thereby, promoting sustained use.

We addressed our hypothesis in a pilot clinical trial that included 27 adolescent CF patients who were introduced to KiOAPP. Here, we present analyses on usage characteristics, product satisfaction, and clinical effects for 25 of them whose questionnaire data were available at the end of the study.

## Methods

### The KiOAPP Mobile Health App

The mHealth app (KiOAPP) was developed by a German nonprofit society for adolescents and young adults after solid organ transplantation [18]. The app is available free of charge in iOS and Android app stores. It offers the following features (also see Multimedia Appendix 1):

- Individualizable medication reminder, including a bar code scanner introduction aid and an audio reminder system (medication reminder function);
- Diary functions for recording daily vitals and personal observations (diary function);
- Communication platform for transmitting information to the physician (contact function); and
- Individualizable user surface (design function).

The integration of a bar code scanning function, allowing capturing bar codes on medication boxes as they are in use in Germany, facilitates the inclusion of a large number of different medications typical in CF into an individual mobile phone-based medication and medication reminder plan. It obviates the need for time-consuming preparation of an entry, which might deter users from this function. We considered the diary function and communication venue useful as they offer day-to-day documentation of symptom load, which adolescents need to learn to assess by themselves when taking over responsibility for their disease from their parents and offers the possibility to discuss changes with the attending physician. We considered the medication plan to provide independence from parental oversight and facilitate self-management of adolescents with CF, thereby providing support in the conflict between independence and disease investment.

### Study Population

Patients were eligible for inclusion into the interventional study via diagnosis of CF and age 12 to 24 years. The Hannover CF center routinely tends to approximately 400 patients with CF across all ages, of whom approximately 150 were of the required age during the recruitment period of the study. All CF patients at our center received a flyer explaining the app and the trial by email and handout upon their clinical appointments at the CF center. They were asked whether they wanted to participate in the study at their next and the consecutive center appointment, leading to a recruitment period of 9 months without randomization and recruitment of 27 patients. Of these patients, 2 were not willing to follow-up via questionnaires after the 3-month intervention period, leading to datasets of 25 participants for analysis at the completion of the study. Not all patients answered all items in all questionnaires. Numbers of patients who replied are indicated in the respective analyses and figures. For analyses on the effects of app use on forced expiratory volume in 1 second (FEV1) and body mass index (BMI), which were available for 25 of the 27 participants who had utilized the app, a control group of 25 CF patients who had not participated in the app study was randomly selected from all CF patients at our center, 2 years after the study. For each study participant, we selected 1 CF patient of similar age, sex, FEV1, and BMI values at the time our interventional study had started.

### Study Design

The study was approved by the local ethics committee (#2826-2015). Upon consent, patients were introduced to the mHealth app, including an explanation of its different functions and the possible usefulness of these function to the everyday treatment burden and self-assessment necessary for patients with CF. At the same time point, all participants were assessed for life satisfaction via FLZ questionnaire (questionnaire on life satisfaction [Fragebogen zur Lebenszufriedenheit]; Multimedia Appendices 2-4), FEV1, and BMI. Within the next week, 4 and 8 weeks later, participants were contacted by phone to ensure installment and understanding of the app. At week 4 and 8 post installment, we systematically assessed app usage and satisfaction via questionnaire III (Multimedia Appendix 5). At these time points, not all participating patients were contactable via phone, leading to 21 (4 weeks) and 20 (8 weeks) phone interviews. Life satisfaction (Multimedia Appendices 2-4), app usage (questionnaire I, *Systems Usability Scale*, Multimedia Appendix 6) and satisfaction (questionnaire II, Multimedia Appendix 7), and product quality (AttrakDiff questionnaire) were consecutively assessed by questionnaires 3 months after recruitment at the observation endpoint, with patients answering paper and Web-based questionnaires during their waiting period for an ambulatory appointment, and interrogation about open questions or uncertainties by the physician during that appointment.

Lung function and BMI were assessed in 3-monthly intervals as part of routine care for the study group and the control group. We compared lung function and BMI of the 25 study subjects who provided questionnaire data at the end of the study and matched 25 control subjects after 3 months, 1 year, and 2 years post inclusion to investigate a possible time-dependent effect after using the app, as lung function and BMI are clinical parameters known to take some time to reflect changes induced by clinical interventions [20].

### Questionnaires

When possible, the authors chose well-established questionnaires which have been evaluated regarding reliability, validity, and reproducibility. Although the authors equally felt that for description of usage characteristics and satisfaction with the app, none of the established test systems offered appropriate content and thus self-designed 2 additional questionnaires, which thus lacked standardized assessment of their test qualities.

The established questionnaires were selected by 4 of the coauthors (HN, UVA, AMD, and KP; with a combined CF expertise of >95 years), each contributing unique backgrounds (social worker, medical doctor and informatician, CF specialist, and medical student within the age range of the target population). The same authors co-designed 2 questionnaires (Multimedia Appendices 5 and 7). After incorporation of feedback to these self-designed questionnaires by our multidisciplinary CF team, including another social worker, a psychologist, a data manager, a dietician, and a physiotherapist, the questionnaires were finalized.

#### App Usage and Satisfaction

App usage and satisfaction were assessed by 3 questionnaires. Questionnaires I (standardized *system usability scale* questionnaire [21], see Multimedia Appendix 6) and II (self-designed questionnaire, see Multimedia Appendix 7) aimed at assessing user behavior. These questionnaires were handed out after the intervention, that is, 12 weeks after the introduction to the mHealth app, during a regular outpatient visit. Questionnaire III (self-designed questionnaire, see Multimedia Appendix 5) was used during telephone interviews with participants, 4 and 8 weeks after the introduction to the mHealth app.

#### Product Qualities of the KiOAPP

Qualities of the KiOAPP were assessed by the AttrakDiff questionnaire, a Web-based questionnaire [22] 12 weeks after the introduction to the mHealth appl during a regular outpatient visit. AttrakDiff is a well-established tool for assessing a product’s usability and design. [23-25]. To this end, it differentiates between pragmatic and hedonic qualities. Usefulness and usability are aspects contributing to pragmatic quality, whereas features that help fulfill emotional needs such as identification with the product, or simply curiosity, are so-called hedonic factors. Both pragmatic and hedonic factors are important for a product’s overall attractiveness. The assessment itself is done via many opposing word pairs, such as *technical* versus *human* or *harmless* versus *challenging*, with participants being asked to rate the product via these word pairs using a scale between −3 and +3.

#### Patients’ Life Satisfaction

Patients’ life satisfaction was assessed by the FLZ questionnaire [26], a well-established questionnaire for the assessment of life satisfaction which offers the additional asset that it includes a CF-specific module and reference population. We assessed life satisfaction at the time of inclusion into the study and 12 weeks after the introduction to the mHealth app during regular outpatient visits. We assessed 3 modules (FLZ): *general life* (see Multimedia Appendix 2), *general health* (see Multimedia Appendix 3), and *CF life* (see Multimedia Appendix 4), each containing 8 categories [26]. The scores for the individual modules were summed up as global scores. Reference populations for the FLZ are CF patients aged 16 to 45 years, n=251 [26]. Analysis of sum scores from study participants aged 12 to 15 years versus study participants aged 16 to 24 years were performed to assess age-specific differences.

### Lung Function

Spirometry was performed according to American Thoracic Society/European Respiratory Society criteria on a PowerCube Body Plethysmograph (Ganshorn). Absolute values were referenced according to Knudson providing percentages compared with a standard reference population and provided for the pertinent time points [27].

### Body Mass Index

BMI measurements were obtained by measurements of weight and height and provided as absolute values.

### Statistical Analyses

Where indicated, arithmetic mean (MW) and standard deviation (SD) were determined for all parameters. Normal distribution was tested with the Kolmogorov-Smirnov test. *P* values were calculated with a one-sample *t* test using the statistical software SPSS (version 23, IBM) where indicated throughout the manuscript. The significance level for all tests is indicated as actual values. Hedge *g* values were used as an alternative evaluation of effect size of the difference between the reference population and our study population by comparing FLZ sum scores between these 2 groups. Cohen *d* values were used as an alternative evaluation of effect size of the intervention by comparing lung function and anthropometry of our study group versus the age and gender-matched control population at given time points.

## Results

### Mobile Health App Usage Characteristics

**Figure 1:**
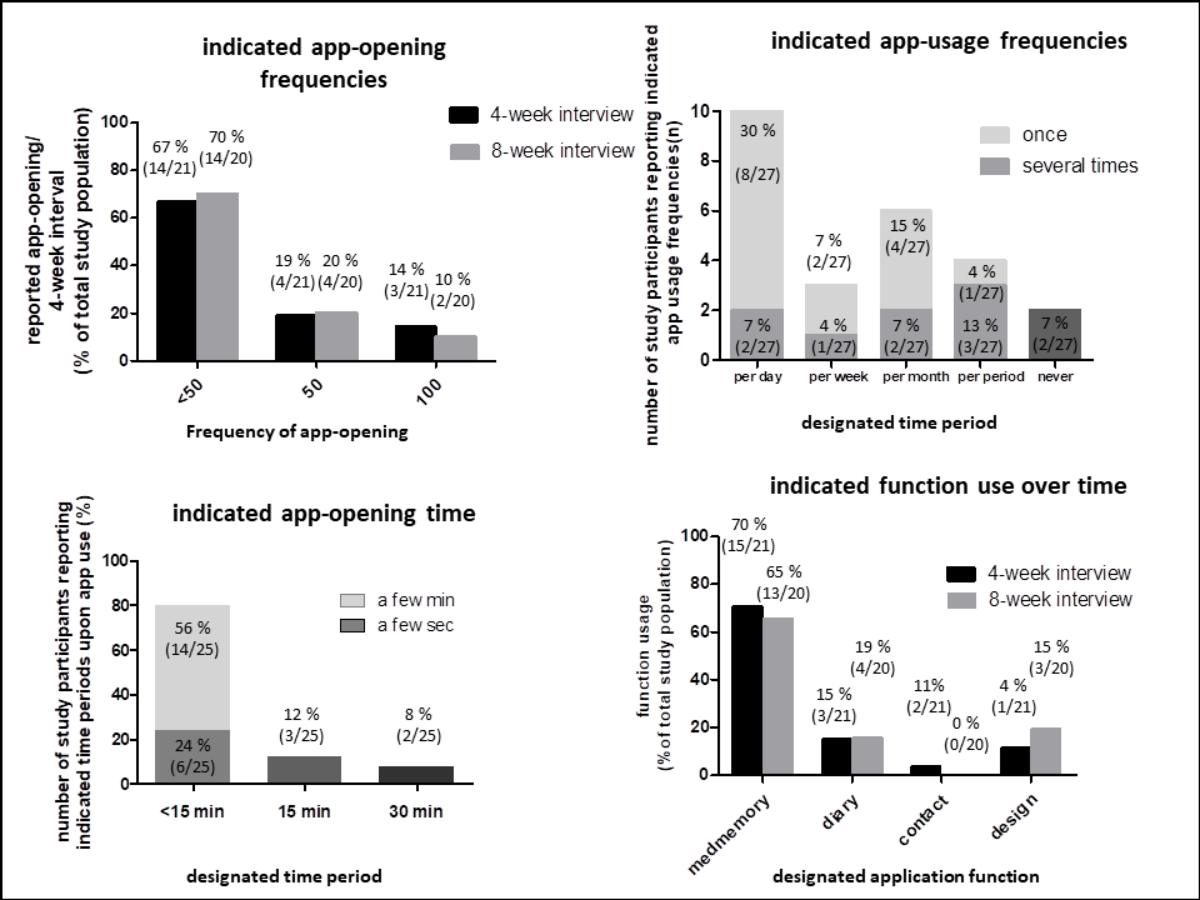
Usage characteristics of the mHealth app. Patients reported number of app-openings per 4 week-interval, estimated overall frequency of app use, reported opening times of app after study completion and use of different app functions. Numbers indicate percentages of participants and overall numbers.

Most of the patients used the app less than 50 times in a 4-week interval, followed by more frequent utilization times. These categories remained stable over the intervention (Figure 1, assessment of question #2 “How often have you used the app in the past 4 weeks?”, questionnaire Multimedia Appendix 3). After completion of the observation period, most of the participants reported that they had used the app several times or at least once per day (Figure 1, assessment of question #2 “How often per day/per month/per three-month have you used the app?”, questionnaire Multimedia Appendix 7). Most participants reported low usage times of a few seconds to minutes per opening of the app (Figure 1, assessment of question #4 “How long have you used the app?”, questionnaire Multimedia Appendix 7).

The medication reminder function was the most frequently used (15/21, 70%, at 4 weeks), followed by the diary function (3/21, 15%, at 4 weeks), the contact function (1/21, 4%, at 4 weeks), and the design function (2/21, 11%, at 4 weeks) (Figure 1, assessment question #1 “Which function of the app have you used in the last 4 weeks?”, questionnaire Multimedia Appendix 5).

Use of the medication reminder function declined between time points from 70% (15/20) at 4 weeks to 65% (13/20) at 8 weeks (Figure 1). Use of the diary function was much lower but remained almost stable at 4 and 8 weeks (3/21 or rather 3/20). The contact function and the design function were both used by only a very limited proportion of participants (Figure 1). Upon examining user patterns, we identified users who (1) used all app functions and reduced use of the medication reminder function over time; (2) used all apps at a steady level; and (3) used only the medication reminder function and reduced its use over time.

### User Ratings: Continuation of Use and Perceived Usefulness

**Figure 2:**
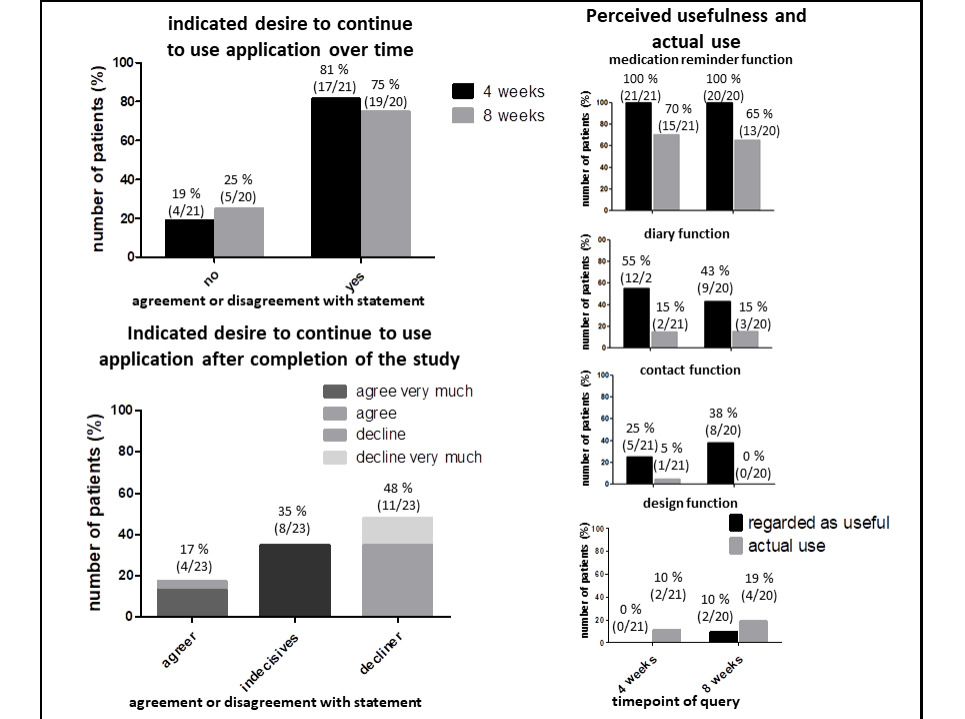
Continuation of use and perceived usefulness versus actual use of mobile health (mHealth) app. Patients reported wish to continue to use app after 4 and 8 weeks and after completion of the study. Patients were queried for perceived usefulness and actual use of different mHealth functions: medication reminder function, diary function, contact function and design function. Perceived usefulness (black bars) was plotted versus reported usage (grey bars). Numbers indicate percentages of participants and overall numbers.

After 4 and 8 weeks, most of the participants wanted to continue to use the app (17/21, 81%, at 4 weeks; 15/20, 75%, after 8 weeks) (Figure 2, assessment question #10 “Can you image to use the app after finishing the study?”, questionnaire Multimedia Appendix 5).

After the completion of the study, not all participants answered the items on their desire to continue to use the app, leading to 23 datasets. Also, the number of participants who wanted to continue to use the app declined to only 17% (4/23) of participants who wanted to continue using the app (Figure 2, sum of *agree very much* and *agree*, see assessment question #1 of the questionnaire in Multimedia Appendix 6) with a large proportion being indecisive (8/23, 35%), and 48% (11/23) of participants declining further usage (Figure 2, sum of *decline* and *decline very much*), suggesting a critical window of attrition between 8 and 12 weeks.

When asked which functions were regarded as useful, results for the medication reminder function were most similar to the actual use of this function (Figure 2, 100% (21/21) perceived usefulness vs. 70% (15/21) actual use at 4 weeks, 100% (20/20) perceived usefulness vs. 65% (13/20) actual use at 8 weeks, composite figure of 2 questions of questionnaire Multimedia Appendix 5: question #4=perceived usefulness, black bars and question #1=actual use, grey bars). Discrepancies between perceived usefulness and actual use were more pronounced for the diary function and the contact possibility as useful (Figure 2). For the design function, the picture was the opposite: more patients used this function than deeming it useful (Figure 2). Still, the latter 2 functions were used and deemed useful only by a small proportion of participants. Perceived usefulness remained stable over the 8-week observation period, with only the design function possibly being regarded more useful after prolonged usage (Figure 2).

### User Ratings: Operability, Hedonistic Qualities, and Attractiveness

**Figure 3:**
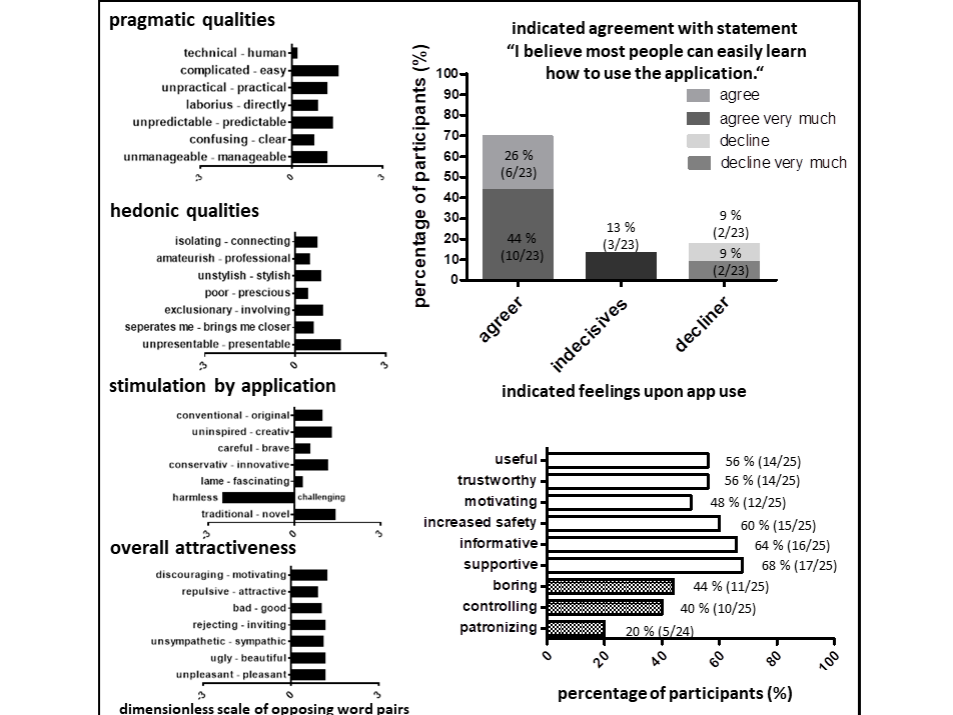
User ratings assessing operability, hedonic qualities, and attractiveness of mobile health app. Pragmatic, hedonic qualities, and stimulation by app as well as overall attractiveness were assessed by the AttrakDiff test. Additionally, participants were queried by questionnaire for usability and for terms describing their feeling upon app use. Numbers indicate percentages of participants and overall numbers.

We used a subtest of the Web-based questionnaire, AttrakDiff (*opposing word pairs*), which assesses product operability (pragmatic quality, Figure 3), hedonic quality (Figure 3), *stimulation of the user by the app* (Figure 3), and overall attractiveness (Figure 3) of the mHealth app to further our understanding of the attitudes which might underlie the attrition we observed. Additionally, satisfaction of operability was assessed after 12 weeks of study participation by direct questionnaire. The overall good satisfaction with operability characteristics of the app by the system’s usability scale [21] (Figure 3, assessment of question #7; questionnaire, Multimedia Appendix 6) was confirmed by results from the AttrakDiff subtest *pragmatic quality* (Figure 3, first graph from top, mean scores for opposing word pairs “as indicated, from top to bottom: 0.2, 1.5, 1.2, 0.9, 1.4, 0.8, 1.2; mean overall score for all 7 word pairs =1.0).

Comparing user ratings of hedonic qualities (Figure 3, second graph from top: mean scores for opposing word pairs as indicated, from top to bottom: 0.8, 0.5, 0.9, 0.4, 0.9, 0.6, 1.5; mean overall score for all 7 word pairs=0.8) and *stimulation* (Figure 3, mean score overall 0.5) with ratings for operability (Figure 3, third graph from top, mean scores for opposing word pairs as indicated, from top to bottom: 1.0, 1.3, 0.6, 1.2, 0.3,−2.5, 1.4, mean overall score for all 7 word pairs=0.5) and overall attractiveness (Figure 3, fourth graph from top, mean for opposing word pairs as indicated, scores from top to bottom: 1.3, 0.9, 1.1, 1.2, 1.2, 1.2, 1.2; mean overall score for all 7 word pairs=1.2), participants attributed more negatively connoted words for hedonic qualities and *stimulation*. It was most pronounced for the area of *stimulation* (Figure 3), attributable mainly to the negative ratings for the word pair *harmless* versus *challenging*, where participants viewed the app strongly *harmless* rather than *challenging*. Overall attractiveness was perceived most positively compared with the 3 other areas (Figure 3).

### Patients Feel Supported by Mobile Health App Use

Upon completion of the 3-month observation period, we questioned the perceived usefulness of the app in supporting better therapeutic adherence, trustworthiness, motivation, promotion of safety, the informative quality and support by the app, boredom, perceived control, and paternalistic quality exerted by the app (Figure 3, questions #7-14, questionnaire Multimedia Appendix 7). Again, not all participants completed all items, leading to 23 to 25 analyzable items, as indicated in Figure 3. The highest positive score was noted for feeling supported (17/25, 68%), followed by a feeling of being informed by the app (16/25, 64%), and a feeling of increased safety (15/25, 60%). Motivation (13/25, 52%), trust (14/25, 56%), and usefulness of the app for therapy adherence (14/25, 56%) were also perceived positively by more than half of the study participants. Fewer patients reported feelings of paternalism (5/24, 20%) or control (10/25, 40%) by the app, or boredom (11/25, 44%). These results suggest that most of the patients perceived the app positively and supportive of therapy adherence.

### Mobile Health App Use Fails to Modulate Weighted Satisfaction With Life and Health

To obtain results on our study populations’ satisfaction with life and health, we assessed the 3 FLZ modules *general life* (questionnaire Multimedia Appendix 2), *health life* (questionnaire Multimedia Appendix 3), and *CF life* (questionnaire Multimedia Appendix 4) in our study group (aged 12-24 years) before and after the mHealth app intervention. The sum scores for different age subgroups of our study group (12-15 years vs 16-24 years) for the 3 domains *general life*, *health life*, and *CF life* revealed no statistically important differences from the results we observed for the overall study population of 12 to 24-year-olds (Table 1).

Our patients showed higher sum scores than the published reference population for weighted satisfaction in all 3 modules at the beginning and end of the observation period, particularly for general life and CF life. Similarly, our study population received higher scores in several subdomain scores. Comparison by Hedge *g* values supports these conclusions, as these attained medium to large effect sizes for most parameters calculated (Table 2). We attribute these differences to (1) inclusion of older patients who have worse health status, since CF is a chronic progressive disease, and (2) the fact that this cohort was sampled >15 years ago [26], when CF patients had worse health status in general. Calculating *P* values for the observed differences never revealed *P*<.05, most likely because of the large standard deviations of the sum scores, and the subitem scores of the FLZ questionnaires [26]. However, the differences in these scores between the study population and the reference population were not a major research question of the study we present here, as we aimed to focus on changes induced by the usage of the mHealth app. In that respect, changes in the difference between our study population and the reference population might indicate effects of mHealth app usage, though, as we elaborate below.

**Table 1:** Questionnaire on life satisfaction (FLZ) sum scores weighted satisfaction for the modules general life, health life, and Cystic Fibrosis life for the complete study group aged 12 to 24 years, and subgroups of 12 to 25 years and 16 to 24 years.

Modules	Study population (age 12-24 years), sum scores		Study population (age 12-15 years), sum scores		Study population (age 16-24 years), sum scores	
	At inclusion	After completion	At inclusion	After completion	At inclusion	After completion
General life	60.04	58.4	62.33	63.6	58.57	54.09
Health life	73.86	72.38	79.89	74.5	70	70.45
Cystic fibrosis life	71.52	67.42	84.6	75.2	63.07	60.36

**Table 2:** Questionnaire on life satisfaction (FLZ) sum scores weighted satisfaction for the modules general life, health life, and Cystic Fibrosis (CF) life, the health life subitems ability to relax and audition/vision and the CF life subitems feeling of being needed/appreciated, understanding/integration of therapy, and lack of handicap by CF of our study population.

Modules and subitems		Reference population (age 16-45 years, n=251), sum scores	Study population (age 12-24 years, n=25), sum scores		Hedge *g* value^a^		*P* value^a^	
			At inclusion	After completion	At inclusion	After completion	At inclusion	After completion
**Modules**								
	General life	46	60.04	58.4	0.44	0.38	.05	.09
	Health life	61.67	73.86	72.38	0.30	0.25	.11	.17
	CF^b^ life	56.01	71.52	67.42	0.41	0.28	.08	.15
**Subitem for the module *health life***								
	Ability to relax	4.84	7.26	7.25	0.51	0.40	.02	.05
**Subitems for the module *CF life***								
	Respiration	4.56	6.5	8.1	0.24	0.44	.08	.05
	Feeling of being needed/appreciated	5.63	9	8.3	0.61	0.48	.02	.12
	Lack of handicap by CF	4.73	9.96	9.4	0.74	0.67	.02	.02
	Understanding/integration of therapy	5.92	8.13	7.7	0.35	0.29	.06	.12

^a^Reference population versus study population.

^b^CF: cystic fibrosis.

Hedge *g* values were calculated to determine the effect size of the intervention by comparing the designated sum scores of the reference population versus the scores of our study population at inclusion and after completion. *P* values were calculated with a 1-sample *t* test between the sum scores of our study population (all participants, ie, age 12-24 years) versus the published reference population of the FLZ questionnaire (age 16-45 years).

The sum scores for *general life* remained the same during the observation period (60.04 vs 58.4 points), whereas the sum scores for *health life* and *CF life* declined during the observation period (73.86 vs 72.38 points and 71.52 vs 67.42 points, respectively, Table 2). These changes failed to attain statistical significance, suggesting that the mHealth app use did not modulate these general domains.

Compared with the published reference population, the *health life* subitem *ability to relax* was significantly higher in our study population at the beginning of the observation period, but not after the intervention (Table 2; *P* value .02 vs .05). In the domain *CF life*, the subitems *feeling of being needed/appreciated* and *lack of handicap by CF* were significantly higher at the beginning of the observation period, compared with the published reference population (*P* value .02 and .02, respectively), which remained significant after the study period for the subitem *lack of handicap by CF* (*P* value .01), suggesting stabilization of this subitem by the app use. The CF-specific subitem *respiration* was improved in our study population after the intervention, compared with values obtained by our study population before app use was assessed (6.5 points before intervention and 8.1 after intervention; Table 2). This increase was not statistically significant for our study population, yet the score was significantly better before and after the intervention, compared with the published reference population (4.56 points reference population vs 8.1 study population before intervention; *P* value .01; 4.56 points reference population vs 6.5 points study population after intervention; *P* value .05; Table 2), again suggesting stabilization of this value by the app use. The subitem *understanding/integration of therapy*, however, was not significantly different from the published reference population either before or after study participation (Table 2).

### Lung Function and Body Mass Index Are Not Affected by Mobile Health App Use

Upon inclusion, the study participants had a mean age of 16 (SD 3) years, a mean FEV1 of 84% (SD 25%), and a mean BMI of 20 (SD 3) kg/m^2^. The control group had a mean age of 15 (SD 3) years, a mean FEV1 of 85% (SD 22%), and a mean BMI of 19 (SD 3) kg/m^2^, which did not differ significantly from the study participants (Table 3). Comparisons of our study group with the matched control group by *P* value or Cohen *d* value to gauge effect sizes of the intervention revealed no differences at 3 months (mean FEV1 for both groups 83%, SD 25% and 22%; Table 3), 1 year (mean FEV1 87%, SD 24% vs 86% SD 23%; Table 3), or 2 years (mean FEV1 79%, SD 25% vs 81%, SD 27%; Table 3]. Comparison of the study group and the control group revealed a small effect size by Cohen *d* value on BMI directly after the study (study group 20 kg/m^2^, SD 3 kg/m^2^; control group 19 kg/m^2^, SD 3 kg/m^2^; Cohen *d* value 0.33) but no differences as per Cohen *d* values or *P* values at the other time points, indicating no consistent effect on somatic parameters.

**Table 3:** Age, forced expiratory volume in 1 second (% Knudson), and body mass index at inclusion of study subjects, and matched controls, 3 months, 1 year, and 2 years post inclusion.

Controls		Study subjects, mean (SD)	n^a^	Matched control group, mean (SD)	n^a^	*P* value	Cohen *d* value^b^
**Prestudy/inclusion (D0)**							
	Age (years)	16 (3)	25	15 (3)	25	.93	—^c^
	FEV1 (%)	84 (25)	25	85 (22)	25	.85	—
	BMI^d^ (kg/m^2^)	20 (3)	25	19 (3)	25	.37	—
**Poststudy (D0 plus 3 months)**							
	FEV1 (%)	83 (23)	25	83 (23)	25	.73	0
	BMI (kg/m^2^)	20 (3)	24	19 (3)	25	.42	0.33
**1-year follow-up (D0 plus 12 months)**							
	FEV1 (%)	87 (24)	25	86 (23)	25	.91	0.042
	BMI (kg/m^2^)	20 (3)	23	20 (2)	25	.77	0
**2-year follow-up (D0 plus 24 months)**							
	FEV1 (%)	79 (25)	21	81 (27)	24	.38	0.08
	BMI (kg/m^2^)	20 (3)	21	20 (2)	23	>.99	>99

^a^Number of available measurements.

^b^Study subjects versus matched control group.

^c^Not applicable.

^d^BMI: body mass index.

Numbers of analyzable data points changed throughout the study because of missing values stemming from routine clinical care. Mean values were calculated for percentage FEV1 and BMI across the study group versus the control group. *P* values were calculated with a 1-sample *t* test between the mean scores of our study group (n=25) versus the control group (n=25). Cohen *d* values were calculated to determine the effect size of the intervention by comparing the FEV1 and BMI values between the study group and the matched control group at the given time points.

## Discussion

### Principal Findings

The medication plan and reminder were the functions used most frequently and perceived most useful (Figures 1 and 2). The diary function and communication venue were used much less frequently, with a larger discrepancy between actual use and perceived usefulness (Figure 1). Finally, the design function was used most infrequently and perceived as of little use. These results might reflect the fact that the desire for independent self-management typical of adolescents was indeed addressed by the KiOAPP medication plan and reminder. Country-specific incorporation of a bar code scanning function, which allows quick incorporation of a large number of different medications typical for CF into the medication plan—an asset for a patient population under chronic time shortage—might have particularly supported the use of this app function. Lesser use of the diary and the communication functions could have been influenced by the fact that the adolescents did not consider these support measures important or that the app did not meet their demands; the discrimination of which probably needs a more qualitative approach to improve our understanding of which measures might further improve disease investment and communication for this age group.

The results indicate that the mHealth app is perceived attractive, useful, and supportive. Nevertheless, upon evaluation over time, we observed attrition in use, similar to other studies [9,10], and, more pronounced, low willingness for continued app usage (Figures 1 and 2). We can only speculate about the reasons for the sharp decline in continued willingness to use the app. Perceived usefulness did not match actual usage of some of the app’s features, possibly because of competing support measures (ie, well-established phone and email contacts in place at our CF center vs the new app contact possibility). Parental (over-) involvement, which did not leave room for adolescents’ involvement, and the well-known developmental challenge of low disease involvement during adolescence [4-6] might have also contributed. Finally, social desirability might have also played a role in the questionnaire responses, leading to overreporting of the perceived usefulness of certain features, with those features the participants actually found supportive likely achieving better matches of perceived usefulness and actual use. Moreover, the least popular function of the app, that is, the design function, only contains 3 options, which might have contributed to its low attractiveness, corroborated by the low ratings in hedonic and *stimulation* qualities we identified via the AttrakDiff questionnaires.

Although we initially thought that the design function could improve adolescents’ acceptance and sustained use of the app, the unfavorable usage characteristics of this function suggest that the KiOAPP addressed these aspects only insufficiently. By assessing opposing word pairs in the AttrakDiff questionnaires, we identified the domain of hedonic qualities and *stimulation* as possible areas to address when aiming to reduce attrition. In the area of hedonic qualities, the AttrakDiff assesses the construct *self-identification* with the product assessed. This area achieved lower ratings compared with pragmatic qualities and overall attractivity (Figure 3). The least positive scores in the 3 subdomains of the word pair test of the AttrakDiff questionnaire were achieved in the area *stimulation*, particularly because of the app being perceived as *harmless* rather than *challenging* (Figure 3). On those lines, *challenges* might improve the motivation of our target population to continue using the app. Our results suggest that adding *gamification* approaches [28], more sophisticated design functions improving *stimulation*, or chat functions improving *self-identification* might be useful to increase app use adherence.

Finally, our results suggest that use of the mHealth app might stabilize satisfaction with the CF-specific item *lack of handicap by CF* and *respiration* in our study group (Table 2), but that it nevertheless has no consistent effect on the stringent clinical parameters FEV1 or BMI (Table 3). These exploratory findings are only insufficiently addressed by our approach and, therefore, do not allow definite conclusions on these interactions but instead require more detailed follow-up studies to address the complex interplay of disease support, disease investment, adherence, quality of life, and somatic function.

### Limitations

Our study and comparisons have several limitations. Methodological approaches such as the choice of questionnaires to assess usage characteristics and user experience, as well as the lack of randomization and blinding possibilities, in addition to recruitment difficulties and the short observation period, might also have precluded the identification of more significant effects by the app use.

The quantitative questionnaires we used might not be ideal to assess user experiences in the detail needed to explain some of the discrepancies of our results, for example, concerning the perceived usefulness and actual use of the app’s features (Figure 2), where qualitative approaches, such as structured interviews, might be more informative. On the other hand, more objective measures, such as electronic usage read-outs, might provide more reliable usage characteristics than the patients’ reported usage characteristics we chose to assess [9]. However, such an approach raises particular ethical concerns as it deeply affects patient privacy, possibly affecting recruitment possibilities, and this was therefore not considered worthwhile exploring at this early stage of the app evaluation. Also, about study design, the difficulties in recruitment precluded random assignment of patients willing to participate in the study into 2 groups (intervention vs no intervention), which might have induced a selection bias in participants. Refining our methodological approach in these areas could be important tools to improve results and reduce ambiguity of results in a future clinical trial.

Only one-sixth of the 150 eligible participants could be recruited into the study, despite intensive recruitment efforts, including hiring of additional personnel and a patient information flyer sent and handed out to all eligible patients. This strongly supports the well-known fact that adolescents and young adults are particularly challenging to draw into clinical studies and to motivate for higher disease investment [5,7], the primary reason for our study. The reasons for this low motivation are most likely multifactorial and correspond to similar findings by others pertaining to CF patients [29], with the overall difficulty in motivation of this particular age group figuring prominently among possible causes [5], potentially even more pronounced in those affected by chronic diseases [4-6] because of a strong desire for normality [5,7]. Numerous competing studies, as well as numerous competing mHealth apps, might have also contributed.

Use of an mHealth app is not a blinded intervention. We chose a case-control design for the lung function and BMI comparisons as an attempt to arrive at meaningful comparisons despite the lack of blinding. Although for the FLZ questionnaires, assessing these questionnaires in an additional cohort at our center was not possible because of the amount of time and personnel necessary, as these questionnaires are not part of the clinical routine follow-ups as in the case of FEV1 and BMI. It left us with a reference population which was not age-matched, and thus we refrain from a comparison with the reference population for the changes we observed.

The short observation period, small study size population, and overall good health of our participants render a measurable improvement or stabilization of FEV1, BMI, and weighted life satisfaction due to any intervention difficult to attain [20]. To identify lung function improvements within a CF patient population with a well-preserved FEV1 as we had included, more sensitive lung function measurements, such as wash-out measurements (lung clearance index) [20], might be necessary, which, unfortunately for the age group we recruited, had not yet been implemented in everyday practice at our center at the time of the study. These shortcomings forego any conclusions about the effect of the mHealth app on somatic parameters, for which larger and longer interventional trials with more sensitive lung function methods are needed for this particular age group.

### Comparison With Prior Work

An improved version of the app might serve to improve app adherence. We chose the app we evaluated as we saw its functionalities address several aspects which might improve adherence in our target population. Still, several aspects might have influenced user affinity, and thereby sustained use, and could have thus impacted negatively on the support potential of the KiOAPP.

User-centered approaches to delineate CF-specific needs for mHealth app development suggest that a multifunctional app incorporating different CF-specific functions such education, enzyme dosage calculation, nutrition management, treatment organization, health diary, treatment follow-up, and practical guidelines for treatment [11], in addition to individuality and adaptability, is a key aspect agreed upon by CF patients [30]. Some freely available CF-specific apps [13,16], as well as current app developments [12,13,15,31], include CF-specific functions identified by a user-centered approach, which has been suggested to be crucial to improve acceptance and reduce attrition of app use [32,33]. The app we evaluated lacks CF-specific features which could reduce attrition. In a free-text item in one of our questionnaires, we did receive feedback that CF-specific features, such as a fat calculator or the possibility to receive reminders for physiotherapy and sports, were considered desirable additions to the existing app. However, the number of answers to the free-text item were too few (n=3 total free-text answers) to address them statistically.

Communication with doctors and peers has also been identified as desirable features by others [30]. Considering that some of the cited studies included a significantly older study population compared with our study [11] and user preferences might reflect age-specific needs concerning mHealth apps [9,17], we suggest including adolescent-specific features into existing disease-specific apps to improve mHealth attachment for this difficult-to-attract age group. Our results on perceived product qualities give indications that, apart from the incorporation of CF-specific needs, additional (age-specific) adaptations, including the incorporation of *hedonistic* features typically desired by adolescents and young adults, might constitute a promising approach to provide adolescent-centered assets to improve app attachment in this age group. While the contribution of hedonistic features is not yet fully understood, this is a notion also supported in the literature (see for example the review presented by Diefenbach et al [34]).

Changes in quality of life have been perceived useful to assess effects of medical interventions in CF, as studies have shown that increases in adherence correlate with a more optimistic view of life in CF [33,35]. Improvements in weighted life satisfaction, measured by the FLZ questionnaire, could have thus indicated improved adherence by mHealth app use. However, as we did not observe such effects and, more importantly, did not include a direct measure of adherence, we cannot make such conclusions on this complex interaction.

Considering that the patient population between 12 and 29 years of age constitutes the largest patient population of Germany’s current CF population [1], our results apply to a significant CF patient number and are probably also generalizable to CF patients in that age group beyond Germany, as CF care is very streamlined worldwide. While patients with other chronic diseases face similar challenges during adolescence, due to our results and those of others concerning disease-specific features of mHealth apps, advocating the development of user-centered approaches [32,33], we caution, however, against generalizing our results to the larger population of adolescents affected by chronic disease.

### Conclusions and Outlook

Low contact availability and disease involvement are challenges all physicians face when caring for adolescent patients with chronic diseases [4-6]. We had postulated that offering support in these areas via the mobile phone might improve self-management for this target age, given their high mobile phone affinity. We must acknowledge that at least in the current form, the app we used might not be sufficient to increase disease involvement of adolescents. However, given the relationship of life satisfaction, optimism, and adherence [6,35], we suggest that an improved version of the mHealth app might constitute a promising tool to improve medication and therapy adherence (via the medication reminder function), disease management (via a fat calculator), and disease investment (via an improved diary function, including at-home lung function monitoring), ultimately improving life satisfaction and also clinical well-being.

Our results suggest that only an improved mHealth app, along with an improved clinical trial design, could add proof to the hypothesis that mHealth apps can constitute a suitable tool to improve self-management and therapy adherence in adolescent patients with CF.

## Appendix

Multimedia Appendix 1 Functions of the mHealth app (KiOAPP).

Multimedia Appendix 2 FLZ module on general life satisfaction.

Multimedia Appendix 3 FLZ module on health life satisfaction.

Multimedia Appendix 4 FLZ module on CF life satisfaction.

Multimedia Appendix 5 Self-developed questionnaire III concerning user behavior used in telephone interviews 4 and 8 weeks after introduction to the mHealth app.

Multimedia Appendix 6 Questionnaire I (SUS) concerning user behavior queried after 12 weeks of mobile health app use.

Multimedia Appendix 7 Self-developed questionnaire II concerning user behavior queried after 12 weeks of mHealth app use.

## Abbreviations

BMI: body mass index
CF: cystic fibrosis
FEV1: forced expiratory volume in 1 second
FLZ: questionnaire on life satisfaction (Fragebogen zur Lebenszufriedenheit)
mHealth: mobile health
