# Supplementary material for: Assessment of a Mobile App by Adolescents and Young Adults With Cystic Fibrosis: Pilot Evaluation
Source: JMIR Mhealth Uhealth. 2019 Nov 21;7(11):e12442. doi: 10.2196/12442 (PMC6895868; doi:10.2196/12442)
Supplement: Multimedia Appendix 1 [file mhealth_v7i11e12442_app1.pdf]

Features of the application with screenshots to illustrate use

a)Medication list and plan

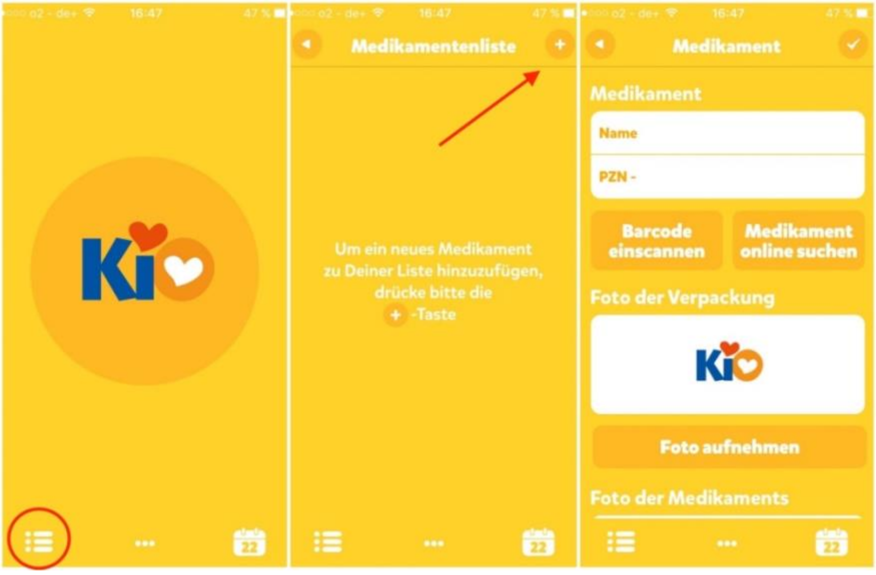

Prepare your own medication list, scan the barcode or use the PZN (German uniform pharmacy number (translation: central pharmacy number) able to unambigously identify every pharmaceutical product licensed in Germany) to search your medication online and take a picture of the medication packaging.

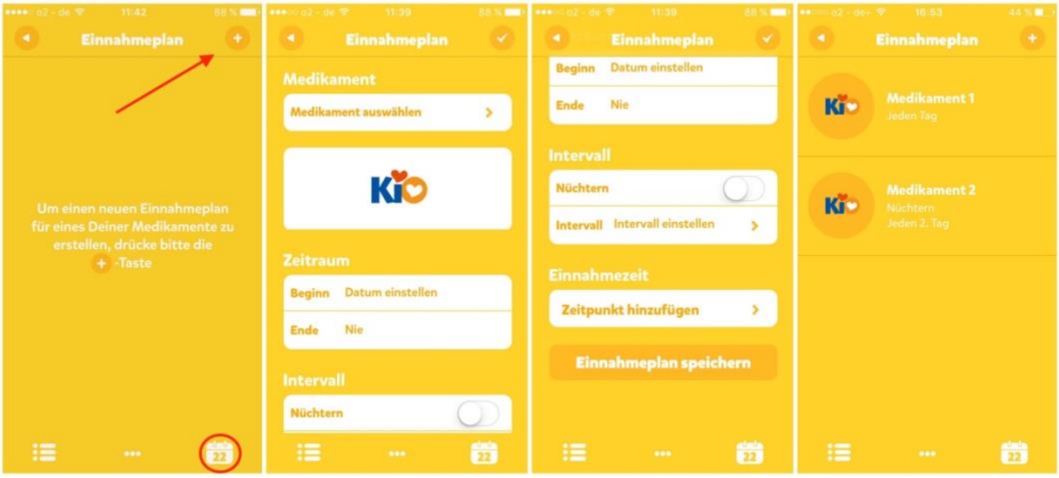

Now choose the medication from your medication list, select the date and the time for medication intake, create your own medication reminder function and have an overview of all your medications.

b)diary function

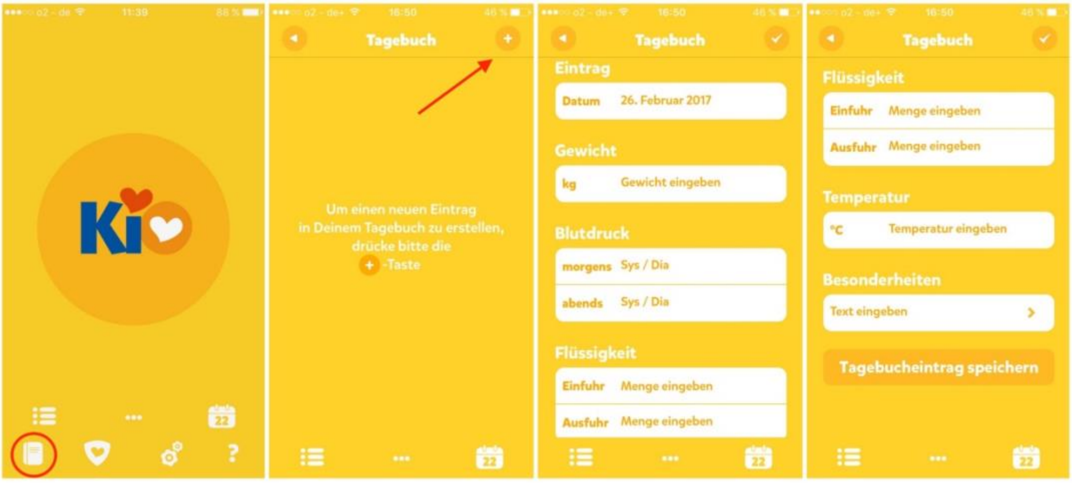

In the diary function you can enter your weight, blood pressure, hydration, temperature and specifics for each day you want.

c)contact function

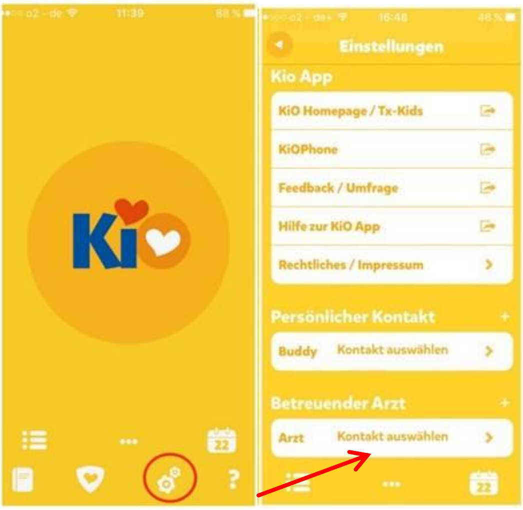

Deposit the email address of your CF doctor for easy contact. Push function allows sending of diary information to designated physician.

d)design function

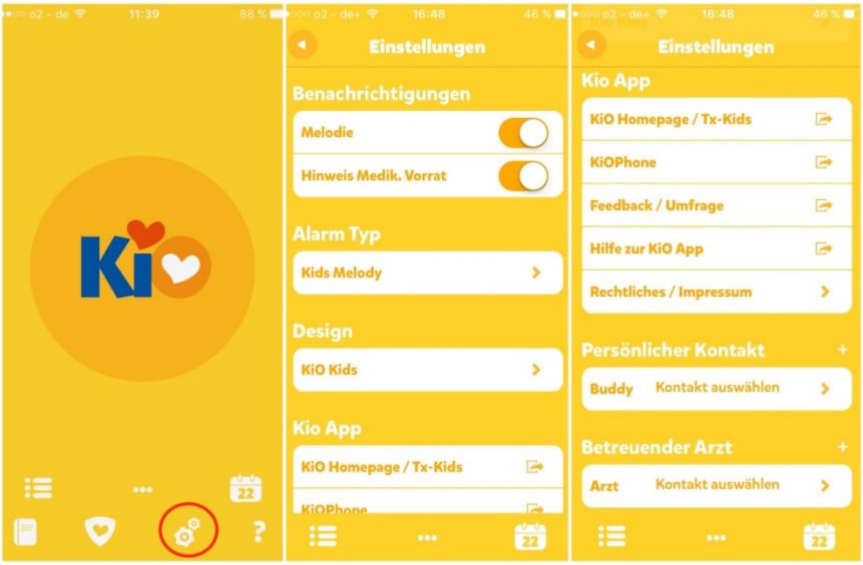

Here you can individualize your application. You can chose your favorite design or alarm type and you can enter the name of your main contact person („buddy“).
