# Supplementary material for: Assessment of a Mobile App by Adolescents and Young Adults With Cystic Fibrosis: Pilot Evaluation
Source: JMIR Mhealth Uhealth. 2019 Nov 21;7(11):e12442. doi: 10.2196/12442 (PMC6895868; doi:10.2196/12442)
Supplement: Multimedia Appendix 2 [file mhealth_v7i11e12442_app2.pdf]

Questionnaire IV/Multimedia Appendix 4: FLZTM questionnaire to assess: *Patients' life satisfaction General*

FLZ Fragen zur Lebenszufriedenheit 1.Allgemeiner Teil

Bei den folgenden Fragen geht es darum, wie **zufrieden** Sie mit Ihrem Leben und mit den einzelnen Aspekten Ihres Lebens sind. Außerdem sollen Sie angeben, wie **wichtig** einzelne Lebensbereiche (z.B. Beruf oder Freizeit) für Ihre Zufriedenheit und Ihr Wohlbefinden sind.

Bitte beantworten Sie **alle** Fragen , auch diejenigen, die scheinbar nicht auf Sie zutreffen: Wenn Sie z.B. keinen Partner haben, können Sie bei der Frage nach der Partnerschaft trotzdem angeben, wie wichtig Ihnen das wäre und wie zufrieden Sie mit der derzeitigen Situation(ohne Partner) sind.

Lassen Sie sich nicht davon beeinflussen, ob Sie sich im Augenblick gut oder schlecht fühlen, sondern versuchen Sie, bei Ihrer Beurteilung **die letzten vier Wochen** zu berücksichtigen.

Bitte kreuzen Sie zunächst an, wie wichtig jeder einzelne Lebensbereich für Ihre Zufriedenheit insgesamt ist. Bevor Sie beginnen, schauen Sie bitte erst alle Bereiche an.

Wie **wichtig** ist für Sie,...

	nicht	etwas	ziemlich<br>wichtig	sehr	extrem
**1) Freunde/ Bekannte**	☐	☐	☐	☐	☐
**2) Freizeitgestaltung/ Hobbies**	☐	☐	☐	☐	☐
**3) Gesundheit**	☐	☐	☐	☐	☐
**4) Einkommen/ finanzielle Sicherheit**	☐	☐	☐	☐	☐
**5) Beruf/ Arbeit bzw. Schule/Ausbildung**	☐	☐	☐	☐	☐
**6) Wohnsituation**	☐	☐	☐	☐	☐
**7) Familienleben/ Kinder**	☐	☐	☐	☐	☐
**8) Partnerschaft/ Sexualität**	☐	☐	☐	☐	☐

Bitte kreuzen Sie nun an, wie zufrieden Sie in den einzelnen Lebensbereichen sind.

Wie **zufrieden** sind Sie mit ....

	un-	eher un-	eher<br>zufrieden	ziemlich	sehr
**1) Freunde/ Bekannte**	☐	☐	☐	☐	☐
**2) Freizeitgestaltung/ Hobbies**	☐	☐	☐	☐	☐
**3) Gesundheit**	☐	☐	☐	☐	☐
**4) Einkommen/ finanzielle Sicherheit**	☐	☐	☐	☐	☐
**5) Beruf/ Arbeit**	☐	☐	☐	☐	☐
**6) Wohnsituation**	☐	☐	☐	☐	☐
**7) Familienleben/ Kinder**	☐	☐	☐	☐	☐
**8) Partnerschaft/ Sexualität**	☐	☐	☐	☐	☐
Wie zufrieden sind Sie mit Ihrem Leben **insgesamt**, wenn Sie alle Aspekte zusammennehmen?	☐	☐	☐	☐	☐

Question about life-satisfaction_1. general section

The following questions address your **satisfaction** with different areas of your life. Additionally, we ask you to estimate the **importance** of these areas in your life (for example professional life or leisure time) for your personal satisfaction and well-being.

Please answer **all** questions, even those apparently not fitting to your situation. If for example you do not have a partner, you can nonetheless estimate how important this could be to you and how satisfied you are with your situation without partner.

Do not let yourself be influenced by acute good or bad feelings but try to evaluate **the last 4 weeks**.

Please firstly designate the importance of the different areas of life. Please look at all areas before starting to fill out the questionnaire.

How **important** are/is…

	**not**	**a little**	**rather important**	**very**	**extremely**
friends/acquaintances					
hobbies					
health					
Income/financial security					
Professional life/work or school/training					
housing situation					
family life/children					
partnership/sexuality					

Please now designate your **satisfaction** with the different aspects of life.

How **satisfied** are you with…

	**unhappy**	**rather unhappy**	**rather happy**	**Very happy**	**extremely happy**
friends/acquaintances					
hobbies					
health					
Income/financial security					
Professional life/work or school/training					
housing situation					
family life/children					
partnership/sexuality					
How happy are you with your life **in general** if you put all the facts together?					
