# Supplementary material for: Assessment of a Mobile App by Adolescents and Young Adults With Cystic Fibrosis: Pilot Evaluation
Source: JMIR Mhealth Uhealth. 2019 Nov 21;7(11):e12442. doi: 10.2196/12442 (PMC6895868; doi:10.2196/12442)
Supplement: Multimedia Appendix 3 [file mhealth_v7i11e12442_app3.pdf]

Questionnaire V/Multimedia Appendix 5: FLZTM questionnaire to assess: *Patients' life satisfaction with Health*

FLZ Fragen zur Lebenszufriedenheit — 2. Gesundheit

Im folgenden ist der Bereich Gesundheit in verschiedene Aspekte unterteilt. Sie sollen auch hier wieder angeben, wie **wichtig** Ihnen einzelne Aspekte sind, und wie **zufrieden** Sie damit sind.
Bitte beantworten Sie **alle** Fragen. Lassen Sie sich nicht davon beeinflussen, ob Sie sich im Augenblick gut oder schlecht fühlen, sondern versuchen Sie, bei Ihrer Beurteilung **die letzten vier Wochen** zu berücksichtigen.
Bitte kreuzen Sie zunächst an, wie wichtig jeder einzelne Aspekt für Ihre Gesundheit ist. Bevor Sie beginnen, schauen Sie bitte erst alle Aspekte an.

Wie **wichtig** ist (sind) für Sie,...	nicht	etwas	ziemlich	sehr	extrem
			wichtig		
**1) Körperliche Leistungsfähigkeit**	☐	☐	☐	☐	☐
**2) Entspannungsfähigkeit/ Ausgeglichenheit**	☐	☐	☐	☐	☐
**3) Energie/ Lebensfreude**	☐	☐	☐	☐	☐
**4) Fortbewegungsfähigkeit (z.B. gehen, Auto fahren)**	☐	☐	☐	☐	☐
**5) Seh- und Hörvermögen**	☐	☐	☐	☐	☐
**6) Angstfreiheit**	☐	☐	☐	☐	☐
**7) Beschwerde- und Schmerzfreiheit**	☐	☐	☐	☐	☐
**8) Unabhängigkeit von Hilfe/ Pflege**	☐	☐	☐	☐	☐

Bitte kreuzen Sie nun an, wie zufrieden Sie in den einzelnen Aspekten sind.

Wie **zufrieden** sind Sie mit Ihrer (Ihrem)....	un-	eher un-	eher	ziemlich	sehr
			zufrieden		
**1) Körperliche Leistungsfähigkeit**	☐	☐	☐	☐	☐
**2) Entspannungsfähigkeit/ Ausgeglichenheit**	☐	☐	☐	☐	☐
**3) Energie/ Lebensfreude**	☐	☐	☐	☐	☐
**4) Fortbewegungsfähigkeit (z.B. gehen, Auto fahren)**	☐	☐	☐	☐	☐
**5) Seh – und Hörvermögen**	☐	☐	☐	☐	☐
**6) Angstfreiheit**	☐	☐	☐	☐	☐
**7) Beschwerde- und Schmerzfreiheit**	☐	☐	☐	☐	☐
**8) Unabhängigkeit von Hilfe / Pflege**	☐	☐	☐	☐	☐

©HeHe München 1990

Question about life-satisfaction_2. health

The following questions address your health, divided into several areas. Again, we ask you to estimate the **importance** of these areas in your life and how **satisfied** you are with them.

Please answer **all** questions.

Do not let yourself be influenced by acute good or bad feelings but try to evaluate **the last 4 weeks**.

Please firstly designate the importance of the different aspects of your health. Please look at all areas before starting to fill out the questionnaire.

How **important** are/is…

	**not**	**a little**	**rather important**	**very**	**extremely**
physical performance					
relaxation ability/balance					
energy/vitality					
locomotion (walking, driving,…)					
vison and hearing					
Lack of anxiety					
absence of symptoms/pain					
independence of help/care					

Please now designate your **satisfaction** with the different aspects of your health.

How **satisfied** are you with your…

	**unhappy**	**rather unhappy**	**rather happy**	**quite happy**	**extremely happy**
physical performance					
relaxation ability/balance					
energy/vitality					
locomotion (walking, driving,…)					
vison and hearing					
Lack of anxiety					
absence of symptoms/pain					
independence of help/care					
