# Supplementary material for: Assessment of a Mobile App by Adolescents and Young Adults With Cystic Fibrosis: Pilot Evaluation
Source: JMIR Mhealth Uhealth. 2019 Nov 21;7(11):e12442. doi: 10.2196/12442 (PMC6895868; doi:10.2196/12442)
Supplement: Multimedia Appendix 4 [file mhealth_v7i11e12442_app4.pdf]

Questionnaire VI/Multimedia Appendix 6: FLZ™ questionnaire to assess: *Patients' life satisfaction with CF*

Fragen zur Lebenszufriedenheit mit Mukoviszidose (FLZ-CF)

Beurteile bitte folgende Aussagen für die letzten 4 Wochen.

Wie **wichtig** ist für Dich,...

	nicht	etwas	ziemlich<br>wichtig	sehr	extrem
1) frei von Atemnot/ Husten zu sein.	☐	☐	☐	☐	☐
2) frei von Bauchschmerzen, Verdauungsbeschwerden zu sein.	☐	☐	☐	☐	☐
3) mit Genuss essen zu können.	☐	☐	☐	☐	☐
4) erholsamen Schlaf finden zu können.	☐	☐	☐	☐	☐
5) den normalen Tagesablauf trotz Therapie aufrechtzuerhalten.	☐	☐	☐	☐	☐
6) Deine tägliche Therapie konsequent durchzuführen.	☐	☐	☐	☐	☐
7) von anderen gebraucht zu werden.	☐	☐	☐	☐	☐
8) von Deiner Umgebung Verständnis für Deine Situation gezeigt zu bekommen.	☐	☐	☐	☐	☐
9) keine Benachteiligung durch Deine Erkrankung zu erfahren.	☐	☐	☐	☐	☐

Bitte kreuze nun an, wie zufrieden Du in den einzelnen Aspekten bist.

Wie **zufrieden** bist Du...

	un-	eher un-	eher<br>zufrieden	ziemlich	sehr
1) mit dem Ausmaß von Atemnot/ Husten.	☐	☐	☐	☐	☐
2) mit dem Ausmaß von Bauchschmerzen/Verdauungsbeschwerden.	☐	☐	☐	☐	☐
3) beim Essen.	☐	☐	☐	☐	☐
4) mit Deinem Schlaf.	☐	☐	☐	☐	☐
5) mit der Integration der Therapie in Deinen Tagesablauf.	☐	☐	☐	☐	☐
6) mit Ihrer Konsequenz bei der täglichen Therapie.	☐	☐	☐	☐	☐
7) mit der Gewissheit, von anderen gebraucht zu werden.	☐	☐	☐	☐	☐
8) mit dem Verständnis Deiner Umgebung für Deine Situation.	☐	☐	☐	☐	☐
9) damit, frei von Benachteiligung zu sein.	☐	☐	☐	☐	☐

® T.Schmitz, L. Goldbeck, Universitätsklinik Ulm und Poliklinik der TU München 2001

Question about life-satisfaction with cystic fibrosis

Please evaluate the following statements for the last 4 weeks.

How **important** is … for you?

	**not**	**a little**	**rather important**	**very**	**extremely**
absence of dyspnoea/coughing					
absence of stomach pains/digestive disorders					
eating and drinking with pleausure					
finding a deep/restful sleep					
keeping the daily routine despite therapy					
doing your daily therapy					
being needed by somebody					
being understood by your environment					
Lack of discrimination because of your illness					

Now tick off how satisfied you are with these aspects.

How **satisfied** are you with …

	**unhappy**	**rather unhappy**	**rather happy**	**quite happy**	**extremely happy**
absence of dyspnoea/coughing					
absence of stomach pains/digestive disorders					
eating and drinking with pleausure					
finding a deep/restful sleep					
keeping the daily routine despite therapy					
doing your daily therapy					
being needed by somebody					
being understood by your environment					
