# Supplementary material for: Assessment of a Mobile App by Adolescents and Young Adults With Cystic Fibrosis: Pilot Evaluation
Source: JMIR Mhealth Uhealth. 2019 Nov 21;7(11):e12442. doi: 10.2196/12442 (PMC6895868; doi:10.2196/12442)
Supplement: Multimedia Appendix 5 [file mhealth_v7i11e12442_app5.pdf]

Questionnaire II/Multimedia Appendix 2: Self-developed questionnaire to assess: *Application usage and satisfaction*

Anonymer Fragebogen zur Nutzung der KIO-App II

Frage #1
Wie häufig hast Du die App benutzt?
(Bitte nur eine Antwort ankreuzen)

[ ] immer
[ ] häufig
[ ] gelegentlich
[ ] selte
[ ] nie

Frage #2
Wie oft hast Du die geschätzt die App aufgerufen?
(Bitte nur eine Antwort ankreuzen)

[ ] einmal am Tag
[ ] mehrfach am Tag
[ ] einmal in der Woche
[ ] mehrfach in der Woche
[ ] einmal im Monat
[ ] mehrfach im Monat
[ ] einmal im gesamten Zeitraum
[ ] mehrfach im gesamten Zeitraum
[ ] nie

Frage #3
Markiere bitte in die untere Zeitleiste mit einem **x**, ab wann Du die App **seltener** als vorher benutzt hast:

I___________________________I_____________________________I

I I I

Anfang der Studie Mitte der Studie Heute

Frage #4
Wie lange hast Du Dich durchschnittlich mit der App beschäftigt, wenn Du sie geöffnet hast?
(Bitte nur eine Antwort ankreuzen)

[ ] sehr lange (eine Stunde und länger)
[ ] lange (halbe Stunde)
[ ] mittel (viertel Stunde)
[ ] kurz (wenige Minuten)
[ ] sehr kurz (wenige Sekunden)

Frage #5
Warum hast Du die App nicht weiter genutzt?
[Freitext]

Frage #6
Was hat Dir bei der App gefehlt?
[Freitext]

Frage #7:
Ich fühlte mich durch die App informiert
(Bitte nur eine Antwort ankreuzen)

[ ] stimme ich zu [ ] stimme ich zum Teil zu [ ] stimme ich nicht zu [ ] weiß nicht

Frage #8:
Ich fühlte mich durch die App bevormundet
(Bitte nur eine Antwort ankreuzen)

[ ] stimme ich zu [ ] stimme ich zum Teil zu [ ] stimme ich nicht zu [ ] weiß nicht

Frage #9:
Ich fühlte mich durch die App unterstützt
(Bitte nur eine Antwort ankreuzen)

[ ] stimme ich zu [ ] stimme ich zum Teil zu [ ] stimme ich nicht zu [ ] weiß nicht

Frage #10:
Ich fühlte mich durch die App kontrolliert
(Bitte nur eine Antwort ankreuzen)

[ ] stimme ich zu [ ] stimme ich zum Teil zu [ ] stimme ich nicht zu [ ] weiß nicht

Frage #11:
Ich fühlte mich durch die App motiviert
(Bitte nur eine Antwort ankreuzen)

[ ] stimme ich zu [ ] stimme ich zum Teil zu [ ] stimme ich nicht zu [ ] weiß nicht

Frage #12:
Ich fühlte mich durch die App gelangweilt
(Bitte nur eine Antwort ankreuzen)

[ ] stimme ich zu [ ] stimme ich zum Teil zu [ ] stimme ich nicht zu [ ] weiß nicht

Frage #13:
Ich fühlte mich durch die App sicherer
(Bitte nur eine Antwort ankreuzen)

[ ] stimme ich zu [ ] stimme ich zum Teil zu [ ] stimme ich nicht zu [ ] weiß nicht

Frage #14:
Ich vertraue der App
(Bitte nur eine Antwort ankreuzen)

[ ] stimme ich zu [ ] stimme ich zum Teil zu [ ] stimme ich nicht zu [ ] weiß nicht

Frage #15:
Die App war für mich hilfreich, meine Therapie besser durchzuführen

[ ] stimme ich zu [ ] stimme ich zum Teil zu [ ] stimme ich nicht zu [ ] weiß nicht

Anonymous questionnaire – utilization of the KIO-App II

Question #1
How often have you used the application?
(please chose one answer)

[]always
[]frequently
[]occasionally
[]rarely
[]never

Question #2
How often per day/per month/per three months have you used the application?
(please chose one answer)

[]once per day
[]several times per day
[]once per week
[]several times per week
[]once per month
[]several times per month
[]once the per period
[]several times per period

Question #3
Please select with “x” on the timeline since when you have reduced your user conduct:

beginning of the study   middle of the study   today

Question #4
How long have you used the application?
(please chose one answer)

[]very long (one hour and more)
[]long (half an hour)
[]middle (15 minutes)
[]short (a few minutes)
[]very short (a few seconds)

Question #5
Why have you stopped using the application? (free text)

Question #6
What have you missed about the application? (free text)

Question #7
I felt informed by the application
(please chose one answer)

[]agree   []partly agree   []disagree   []do not know

Question #8
I felt patronized by the application

(please chose one answer)

[]agree []partly agree []disagree []do not know

Question #9
I felt supported by the application
(please chose one answer)

[]agree []partly agree []disagree []do not know

Question #10
I felt controlled by the application
(please chose one answer)

[]agree []partly agree []disagree []do not know

Question #11
I felt motivated by the application
(please chose one answer)

[]agree []partly agree []disagree []do not know

Question #12
I felt bored by the application
(please chose one answer)

[]agree []partly agree []disagree []do not know

Question #13
I felt safer by the application
(please chose one answer)

[]agree []partly agree []disagree []do not know

Question #14
I trust the application
(please chose one answer)

[]agree []partly agree []disagree []do not know

Question #15
The application was helpful to improve management of my therapy
(please chose one answer)

[]agree []partly agree []disagree []do not know
