# Supplementary material for: Assessment of a Mobile App by Adolescents and Young Adults With Cystic Fibrosis: Pilot Evaluation
Source: JMIR Mhealth Uhealth. 2019 Nov 21;7(11):e12442. doi: 10.2196/12442 (PMC6895868; doi:10.2196/12442)
Supplement: Multimedia Appendix 6 [file mhealth_v7i11e12442_app6.pdf]

Questionnaire I/Multimedia Appendix 1: Systems Usability Scale to assess: *Application usage and satisfaction*

Anonymer Fragebogen zur Nutzung der KIO-App I

Wir möchten Sie/Dich um eine kurze Einschätzung der Handhabbarkeit (Usability) der KIO- App anhand des unten stehenden Fragebogens bitten. Die Befragung ist anonym, vollkommen freiwillig und in 3 Minuten zu bearbeiten. Ihre/Deine Angaben helfen uns, den Nutzen der Anwendung besser einschätzen zu lernen.
Vielen Dank für Ihre/Deine Mithilfe!

Ausfüllhinweis: Bitte markieren Sie einen der fünf Kreise mit einem „X" für eine Antwort. In dem Beispiel rechts ist das 4. Feld markiert, was als Antwort einem „stimme zu" entspräche.	O<br>1<br>lehne sehr ab	O<br>2	O<br>3	X<br>4	O<br>5<br>stimme sehr zu
1. Ich denke, dass ich die Anwendung regelmäßig nutzen möchte.	O<br>1<br>lehne sehr ab	O<br>2	O<br>3	O<br>4	O<br>5<br>stimme sehr zu
2. Ich empfand die Anwendung als unnötig kompliziert.	O<br>1<br>lehne sehr ab	O<br>2	O<br>3	O<br>4	O<br>5<br>stimme sehr zu
3. Ich fand die Anwendung leicht zu benutzen.	O<br>1<br>lehne sehr ab	O<br>2	O<br>3	O<br>4	O<br>5<br>stimme sehr zu
4. Ich denke, dass ich die Hilfe von einem Techniker bräuchte, um die Anwendung nutzen zu können.	O<br>1<br>lehne sehr ab	O<br>2	O<br>3	O<br>4	O<br>5<br>stimme sehr zu
5. Ich denke, dass die verschiedenen Funktionen in der Anwendung gut integriert wurden.	O<br>1<br>lehne sehr ab	O<br>2	O<br>3	O<br>4	O<br>5<br>stimme sehr zu
6. Ich fand, dass es zu viele Widersprüchlichkeiten in der Anwendung gab.	O<br>1<br>lehne sehr ab	O<br>2	O<br>3	O<br>4	O<br>5<br>stimme sehr zu
7. Ich könnte mir vorstellen, dass die meisten Menschen schnell lernen können, wie die Anwendung funktioniert.	O<br>1<br>lehne sehr ab	O<br>2	O<br>3	O<br>4	O<br>5<br>stimme sehr zu
8. Ich empfand die Anwendung als umständlich zu benutzen.	O<br>1<br>lehne sehr ab	O<br>2	O<br>3	O<br>4	O<br>5<br>stimme sehr zu
9. Ich fühlte mich in der Benutzung der Anwendung sicher.	O<br>1<br>lehne sehr ab	O<br>2	O<br>3	O<br>4	O<br>5<br>stimme sehr zu
10. Ich musste viele Dinge erst lernen bevor ich die Anwendung nutzen konnte.	O<br>1<br>lehne sehr ab	O<br>2	O<br>3	O<br>4	O<br>5<br>stimme sehr zu

Anonymous questionnaire – utilisation of KIO-App I

We would like to briefly obtain your assessment of the usability of the KIO-App by the questionnaire below. The questionnaire is anonymous, voluntary and can be accomplished within 3 minutes. Your assessment will help us to understand the usefulness of the application.
Thank you very much for your help.

How to complete the questionnaire: please mark one of the five circles with an X for your preferred answer. In the example to the right, the fourth circle is marked, corresponding to the answer "agree".

	**statement**	**1**	**2**	**3**	**4**	**5**
1	"I think I will use the application regularly."					
2	"I think the application was unnecessarily complicated."					
3	"I think the application was easy to use."					
4	"I think I need a technician's help to use the application."					
5	"I believe that the different functions of the application are well integrated."					
6	"I think that there are too many discrepancies in the application."					
7	"I believe that the most of the people will learn quickly how to use the application."					
8	"I think the application was complicated to use."					
9	"I felt secure using the application."					
10	"I had to learn a lot until I could use the application."					

Legend:
1=decline very much, 2=decline, 3=indecisive, 4=agree, 5=agree very much

J, B. (1996). SUS-A quick and dirty usability scale. Usability Evaluation in Industry. T. B. Jordan PW, Weerdmeester BA, McClelland AL. London, Taylor and Francis.
