# Supplementary material for: Assessment of a Mobile App by Adolescents and Young Adults With Cystic Fibrosis: Pilot Evaluation
Source: JMIR Mhealth Uhealth. 2019 Nov 21;7(11):e12442. doi: 10.2196/12442 (PMC6895868; doi:10.2196/12442)
Supplement: Multimedia Appendix 7 [file mhealth_v7i11e12442_app7.pdf]

Questionnaire III/Multimedia Appendix 3: Self-developed questionnaire to assess: *Application usage and satisfaction*

CF-Transform

Fragen zur Nutzung der KIO-APP nach 4 bzw.8 Wochen

1. Welche Funktionen hast Du in den letzen 4 Wochen genutzt?
   a) Medikamenteneinnahme/Erinnerungsfunktion
   b) Tagebuch
   c) Kontaktmöglichkeit mit dem Arzt
   d) Design
2. Wie oft hast du die App genutzt in den letzten 4 Wochen genutzt?
   a) 100 mal
   b) 50 mal
   c) weniger
3. Hat sich Dein Nutzungsverhalten in den letzten 4 Wochen verändert?
   a) Ja
   b) nein
4. Welche Funktion hältst Du für sinnvoll?
   a) Medikamenteneinnahme/Erinnerungsfunktion
   b) Tagebuch
   c) Kontaktmöglichkeit mit dem Arzt
   d) Design
5. Welche Funktion nutzt du nicht?
   a) Medikamenteneinnahme/Erinnerungsfunktion
   b) Tagebuch
   c) Kontaktmöglichkeit mit dem Arzt
   d) Design
6. Welche Funktion fehlt dir bei der App?
   a) freie Antwort möglich
7. Hast Du jemandem von der App erzählt?
   a) Ja
   b) nein
8. Hast du sie jemandem gezeigt/vorgestellt?
   a) Ja
   b) nein
9. Ist die Erinnerungsfunktion ein Störfaktor in deinem Alltag?
   a) Ja
   b) nein
10. Stellst du dir vor die App nach dem Ende der Studie weiter zu verwenden?
    a) Ja
    b) nein

CF-Transform

Question about the use of the KIO-APP after 4 and 8 weeks

1. Which function of the application have you used in the last 4 weeks?
   a) medication memory

b) diary
c) contact
d) design

2. How often have you used the application in the last 4 weeks?
    a) 100 times
    b) 50 times
    c) Less

3. Has your user conduct changed in the last 4 weeks?
    a) yes
    b) no

4. Which functions do you think are useful?
    a) medication memory
    b) diary
    c) contact
    d) design

5. Which function don’t you use?
    a) medication memory
    b) diary
    c) contact
    d) design

6. Which function do you miss? (free text)

7. Have you told anybody about the application?
    a) yes
    b) no

8. Have you shown anyone the application?
    a) yes
    b) no

9. Is the memory function a disruptive factor in your everyday life?
    a) yes
    b) no

10. Can you imagine to use the application after finishing the study?
    a) yes
    b) no
